# Effect of High-Flow Oxygen on Exercise Performance in COPD Patients. Randomized Trial

**DOI:** 10.3389/fmed.2020.595450

**Published:** 2021-02-19

**Authors:** Konstantinos Bitos, Michael Furian, Laura Mayer, Simon R. Schneider, Simone Buenzli, Maamed Z. Mademilov, Ulan U. Sheraliev, Nuridin H. Marazhapov, Ainura K. Abdraeva, Shoira D. Aidaralieva, Aybermet M. Muratbekova, Talant M. Sooronbaev, Silvia Ulrich, Konrad E. Bloch

**Affiliations:** ^1^Department of Respiratory Medicine and Sleep Disorders Center, University Hospital Zurich, Zurich, Switzerland; ^2^Swiss-Kyrgyz High Altitude Medicine and Research Initiative, Zurich, Switzerland; ^3^Swiss-Kyrgyz High Altitude Medicine and Research Initiative, Bishkek, Kyrgyzstan; ^4^National Center of Cardiology and Internal Medicine, Bishkek, Kyrgyzstan

**Keywords:** COPD, exercise, randomized controlled trial, oxygen therapy, high-flow

## Abstract

**Background:** High-flow oxygen therapy (HFOT) provides oxygen-enriched, humidified, and heated air at high flow rates via nasal cannula. It could be an alternative to low-flow oxygen therapy (LFOT) which is commonly used by patients with chronic obstructive pulmonary disease (COPD) during exercise training.

**Research Question:** We evaluated the hypothesis that HFOT improves exercise endurance in COPD patients compared to LFOT.

**Methods:** Patients with stable COPD, FEV_1_ 40–80% predicted, resting pulse oximetry (SpO_2_) ≥92%, performed two constant-load cycling exercise tests to exhaustion at 75% of maximal work rate on two different days, using LFOT (3 L/min) and HFOT (60 L/min, FiO_2_ 0.45) in randomized order according to a crossover design. Primary outcome was exercise endurance time, further outcomes were SpO_2_, breath rate and dyspnea.

**Results:** In 79 randomized patients, mean ± SD age 58 ± 9 y, FEV_1_ 63 ± 9% predicted, GOLD grades 2-3, resting PaO_2_ 9.4 ± 1.0 kPa, intention-to-treat analysis revealed an endurance time of 688 ± 463 s with LFOT and 773 ± 471 s with HFOT, mean difference 85 s (95% CI: 7 to 164, *P* = 0.034), relative increase of 13% (95% CI: 1 to 28). At isotime, patients had lower respiratory rate and higher SpO_2_ with HFOT. At end-exercise, SpO_2_ was higher by 2% (95% CI: 2 to 2), and Borg CR10 dyspnea scores were lower by 0.8 points (95% CI: 0.3 to 1.2) compared to LFOT.

**Interpretation:** In mildly hypoxemic patients with COPD, HFOT improved endurance time in association with higher arterial oxygen saturation, reduced respiratory rate and less dyspnea compared to LFOT. Therefore, HFOT is promising for enhancing exercise performance in COPD.

**Clinical Trial Registration:** www.ClinicalTrials.gov, identifier: NCT03955770.

## Introduction

Chronic obstructive pulmonary disease (COPD) is a progressive disease characterized by chronic obstruction of airflow related to airway inflammation, remodeling, and parenchymal destruction of the lung (1) and is the fourth leading cause of death worldwide (2). Among patients with COPD, airflow obstruction, hyperinflation and gas exchange impairment leading to dyspnea as well as muscle weakness are important factors contributing to exercise intolerance (3). Pulmonary rehabilitation is an effective means to improve dyspnea, exercise performance, health status and quality of life in this population (1, 4, 5). In particular, endurance training improves the aerobic exercise capacity and peripheral muscle function (6). Low-flow oxygen therapy (LFOT) during constant-load exercise improves muscle oxygenation (7) and exercise endurance time (8) both in COPD patients with and without exercise-induced hypoxemia (9). However, LFOT cannot always assure appropriate oxygenation, does not support ventilation and may lead to uncomfortable drying-out of airways (10).

High-flow oxygen therapy (HFOT) is a novel modality of ventilatory support which provides heated and humidified oxygen-air mixtures at selected FiO_2_ and high flow rates (up to 60 L/min) via a large bore nasal cannula. Physiological studies have shown that HFOT improves oxygenation and at the same time washes out carbon dioxide in the anatomical dead space (11). HFOT has also been shown to reduce the respiratory rate (12, 13) and work of breathing and to induce a slight positive end-expiratory pressure (PEEP, usually around 3 cm H_2_O) supporting oxygenation and upper airway patency (14). Unlike conventional LFOT, HFOT has the advantage to deliver a stable, selectable FiO_2_ (0.21 to 1.0), even during mouth breathing (14). Several randomized trials have suggested potential benefits of HFOT in critically ill hypoxemic patients in comparison to non-invasive ventilation or LFOT (15, 16). In COPD patients with chronic, stable, hypoxemic respiratory failure, a recent randomized trial revealed a positive effect of long-term home HFOT vs. conventional LFOT in terms of symptoms, COPD-exacerbations and hospital admissions (17). In a recent study in COPD patients, HFOT employed during exercise training over the course of a 4-weeks rehabilitation program did not increase endurance time compared to oxygen supplementation via a Venturi mask (18). Further investigations in patients with stable COPD, revealed conflicting results with HFOT in terms of enhancing exercise endurance and definitive conclusions were hampered by the small sample size and methodological weakness of certain studies (19, 20).

The purpose of the current study was therefore to investigate the effect of HFOT on exercise endurance time in patients with COPD compared to LFOT. We choose to administer HFOT with a total flow of 60 l/min, FiO_2_ 0.45, to optimally support ventilation and oxygenation in a clinically feasible way. LFOT at a rate of 3 l/min via conventional nasal cannula was selected as the comparator because this mode of oxygen supplementation has been widely used in clinical practice and research studies (21, 22) and because higher flow rates of cold and dry oxygen may have induced nasal mucosal irritation and discomfort. The main hypothesis was that, in patients with moderate to severe COPD, HFOT improves the constant-load cycling endurance time compared to LFOT.

## Methods

### Study Design

This randomized, crossover trial evaluated the effect of HFOT on cycling endurance time in patients with COPD in comparison to LFOT. After baseline assessments, patients were randomized to a constant-load exercise test under LFOT (oxygen flow 3 l/min via conventional nasal cannula) first, followed by a test under HFOT (total flow 60 L/min, FiO_2_ 0.45, via Optiflow nasal cannula, see below) second, on a different day, or vice versa, with a washout period of at least 1 day. The predetermined settings for LFOT and HFOT were identical for all participants and left unchanged during exercise tests. The study was performed from May to July 2019 at the National Center of Cardiology and Internal Medicine (NCCIM) in Bishkek, Kyrgyzstan. The protocol was approved by the Ethics Committee of the NCCIM (2019-15) and registered at www.ClinicalTrials.gov (NCT03955770). Written informed consent was obtained from all participants.

### Patients

Men and women, 35 to 75 years of age, with COPD diagnosed according to Global Initiative for Obstructive Lung Disease (GOLD) guidelines (1), FEV_1_/FVC <0.7, FEV_1_ 40–80% predicted, resting pulse oximetry (SpO_2_) ≥92%, PaCO_2_ <6 kPa, were included. Exclusion criteria were current long-term oxygen therapy, current heavy smoking (>20 cigarettes per day) and comorbidities such as uncontrolled cardiovascular disease, internal, neurologic, rheumatologic or psychiatric disease that might have interfered with protocol compliance.

### Assessments and Interventions

Baseline evaluations included a medical history, clinical examination, the modified Medical Research Council dyspnea score (23), spirometry, arterial blood gas analysis and a 6-min walk test.

Each patient performed two constant-load cycling exercise tests to exhaustion using HFOT and LFOT on two different days, respectively, according to randomization. The load of the stationary cycle ergometer was set at 75% of the individually estimated maximum work rate using an approach similar to that proposed by Luxton et al. based on studies in 22 COPD patients (18, 24). Thus, we estimated the individual maximal work rate by a regression model fitted to data from a previous study in 134 COPD patients (25) using sex, age (coefficient in men −1.12, in women −0.71) FEV_1_% predicted (coefficient in men 0.76, in women 0.19), body mass index (coefficient in men 1.56, in women 0.97), 6-min walk distance (coefficient in men 0.11, in women 0.14) and a constant (in men 34.8, in women 16.0) into account. After a 2-min resting period on the ergometer, patients started exercise. They were encouraged to maintain a cycling rate of >60 rounds/min for as long as possible until exhaustion. The test was stopped if cycling rate dropped to <40 rounds/min for >10 s and this time was recorded as end-exercise. Additional pre-defined termination criteria included chest pain and ECG changes suggesting cardiac ischemia, uncontrolled arterial hypertension and severe symptomatic oxygen desaturation (SpO_2_ ≤ 80%), among others, according to published standards (26). If the duration of the initial test was <3 or >25 min, the intervention was repeated on another day with adjusted work load to achieve a duration of 3–25 min. This was required in five patients starting with LFOT and eight patients starting with HFOT. During exercise tests, LFOT was provided by a standard nasal cannula (oxygen cannula standard connector, Dahlhausen) at predetermined and fixed flow rate of 3 L/min using an oxygen concentrator (EverFlow, Philips Respironics, providing FiO_2_ >0.95). HFOT was applied by a dedicated large bore nasal cannula (Optiflow+, Fisher&Paykel, New Zealand) with a predetermined, fixed flow rate of 60 L/min, FiO_2_ 0.45, temperature 31°C, using a HFOT device (myAIRVO2, Fisher&Paykel, New Zealand) in combination with five oxygen concentrators (EverFlow, Philips Respironics) connected in parallel to deliver the required oxygen admixture of 18–20 L/min. The predetermined standard settings of LFOT and HFOT were left unchanged during all exercise tests.

A 3-lead ECG, pulse oximetry and respiratory inductance plethysmography were continuously monitored (Alice 5, polygraphy device, Philips Respironics), blood pressure was non-invasively measured by a cuff system. A radial arterial blood sample was drawn in the final 30 s of exercise to measure arterial blood gases (RapidPoint500, Siemens). The endurance time from beginning to end-exercise was recorded. Mean values of ECG-derived heart rate, pulse oximetry, and breath rate from inductance plethysmography were obtained during 30 s at rest, immediately before the start of exercise, during the final 30 s of exercise, and during 30 s over the course of the test with longer endurance at an elapsed time corresponding to the final 30 s of the test with the shorter endurance to compare isotime values between treatments. The heart rate reserve was calculated as the maximal predicted heart rate (i.e., 220-age) minus the observed heart rate. Immediately before and after the test, patients rated perceived dyspnea using the Borg CR10 scale (27) and indicated the comfort of the treatment on a 100-mm visual analog scale ranging from 0 (extremely uncomfortable) to 100 (very comfortable).

### Outcomes and Sample Size Estimation

The primary outcome was the difference in exercise endurance time between tests on HFOT vs. LFOT. Secondary outcomes included heart rate, respiratory rate, pulse oximetry, arterial blood gases, dyspnea sensation and treatment comfort. In patients with COPD, a minimal difference in endurance time assessed by constant-load bicycle ergometry of 75 s (range 46–105 s) or an effect size of >0.36 have been shown to be clinically important (28). Therefore, to detect an improvement in cycling time of 50 s, effect size of 0.36 (corresponding to a SD of 140 s), 64 patients were needed to power the study with 80%, alpha 0.05. Accounting for drop-outs, we aimed to recruit 80 participants.

### Randomization and Masking

Randomization was performed by a study coordinator as per computer generated schedule (using the software MinimPY) (29) minimizing for age (<60 or ≥60 y), sex, FEV_1_% predicted (<60 or ≥60) and 6-min walking distance (<500 or ≥500 m). Patients were explained that two different forms of oxygen therapy would be compared, but no further details were disclosed.

### Data Analysis and Statistics

Data are summarized by numbers and proportions or means ± SD. The primary outcome was analyzed by the intention-to-treat approach, replacing missing values by multiple imputation using chained equations (30). Adjusted treatment effects were computed by incorporating age, sex and FEV_1_ as additional predictors. Secondary outcomes were analyzed according to the per-protocol approach using data from all patients with complete data. Means and 95% confidence intervals (CI) of the unadjusted treatment effects on the primary and secondary outcomes were assessed by mixed effects linear regression analyses including intervention (HFOT, LFOT) and allocation sequence (HFOT first, LFOT first) as covariates. Effect sizes were computed according to Kazis et al. (31) A probability of *P* < 0.05 was assumed as significant.

## Results

**Figure 1:**
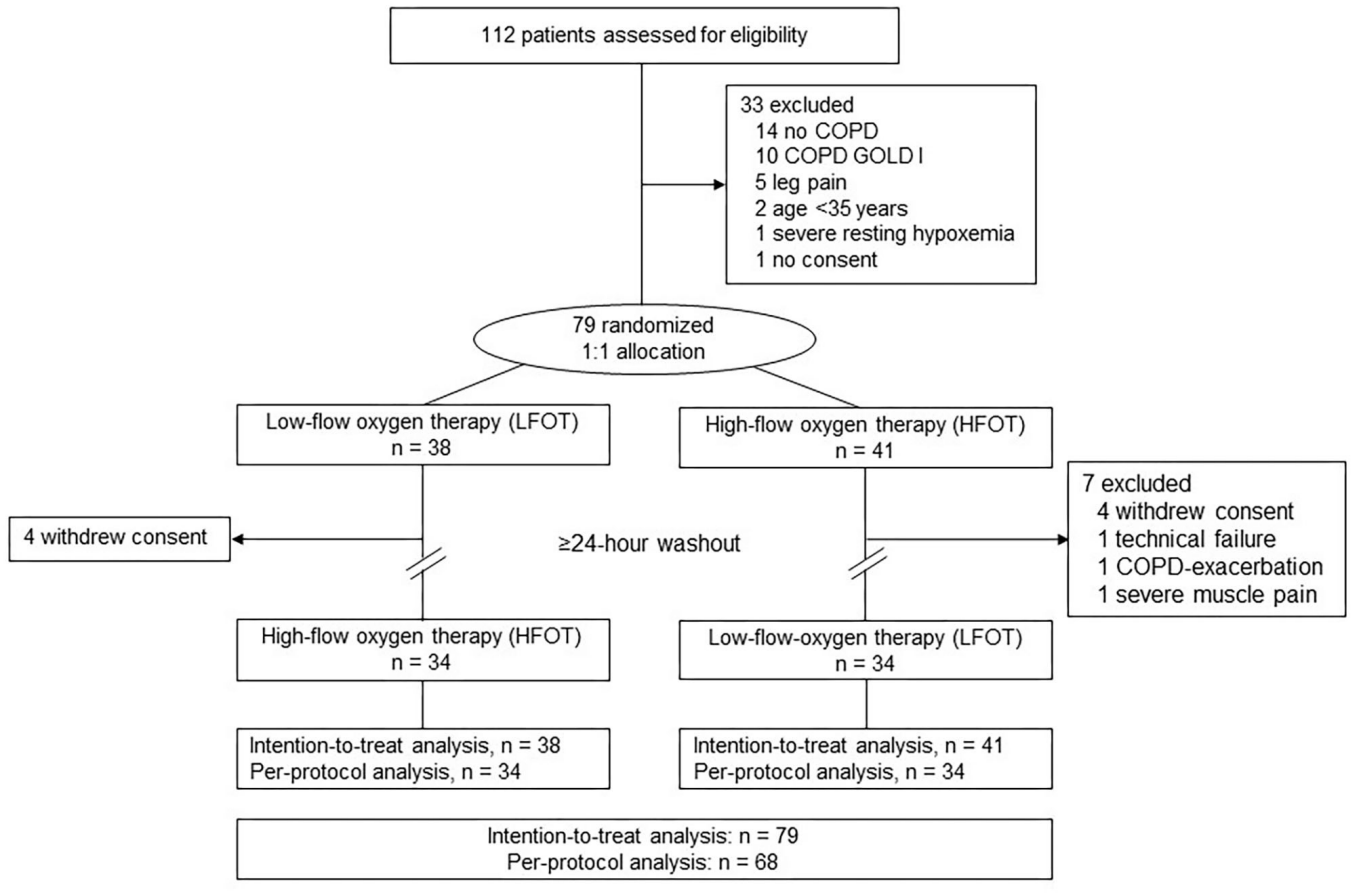
Patient flow in the cross-over trial.

Of 112 screened individuals, 79 were randomized and included in the intention-to-treat analysis (Figure 1). Eleven had to be excluded after performing the first test for various reasons, most commonly because they withdrew consent (Figure 1). Data from 68 patients (34 in each arm) were available for the per-protocol analysis. Patient characteristics were similar between the two arms (Table 1). No relevant adverse events occurred during the study.

**Table 1:** Patient characteristics.

	**Allocation sequence LFOT first *n* = 38**	**Allocation sequence HFOT first *n* = 41**	**All patients *n* = 79**
Sex, m/f	23/15	22/19	45/34
Age, y	57.9 ± 8.6	58.4 ± 9.1	58.1 ± 8.8
Body mass index, kg/m^2^	27.8 ± 5.4	27.4 ± 4	27.6 ± 4.7
FEV_1_,% predicted	63 ± 9.4	63 ± 9.6	63 ± 9.4
COPD GOLD 2/3, *n*	34/4	36/5	70/9
6-minute walk distance, m	483 ± 78	498 ± 71	491 ± 75
Drop in pulse oximetry ≥2% during 6-min walk test, *n* patients	4	11	15
Smoking status			
Current smoker, *n* (%)	11 (28.9)	5 (12.2)	16 (20.3)
Ex-smoker, *n* (%)	9 (23.7)	13 (31.7)	22 (27.8)
Smoking, Pack-years	16 (12.7)	18 (17)	34 (43)
Current medication			
Inhaled beta-adrenergics, *n* (%)	10 (26)	10 (24)	20 (25)
Inhaled anticholinergics, *n* (%)	5 (13)	10 (24)	15 (19)
Inhaled steroids, *n* (%)	9 (24)	9 (22)	18 (23)
Antihypertensives, *n* (%)	6 (16)	6 (15)	12 (15)
Beta-blockers, *n* (%)	0 (0)	2 (5)	2 (3)
Antidiabetiks, *n* (%)	1 (3)	1 (2)	2 (3)
Acetylsalicylic acid, *n* (%)	4 (11)	2 (5)	6 (8)
Arterial blood gas analysis (at rest, ambient air)			
pH	7.41 ± 0.03	7.40 ± 0.02	7.40 ± 0.02
PaO_2_, kPa	9.3 ± 0.9	9.5 ± 1.1	9.4 ± 1.0
PaCO_2_, kPa	5.5 ± 0.5	5.4 ± 0.5	5.5 ± 0.5
${\text{HCO}}_{3}^{-}$, mmol/L	25.2 ± 2.5	24.6 ± 1.9	24.9 ± 2.2
Lactate, mmol/L	0.97 ± 0.32	1.06 ± 0.36	1.02 ± 0.34
Glucose, mmol/L	6.00 ± 0.93	5.72 ± 0.89	5.84 ± 0.91

*Means ± SD, numbers (%). LFOT, Low-flow oxygen therapy; HFOT, high-flow oxygen therapy*.

**Figure 2:**
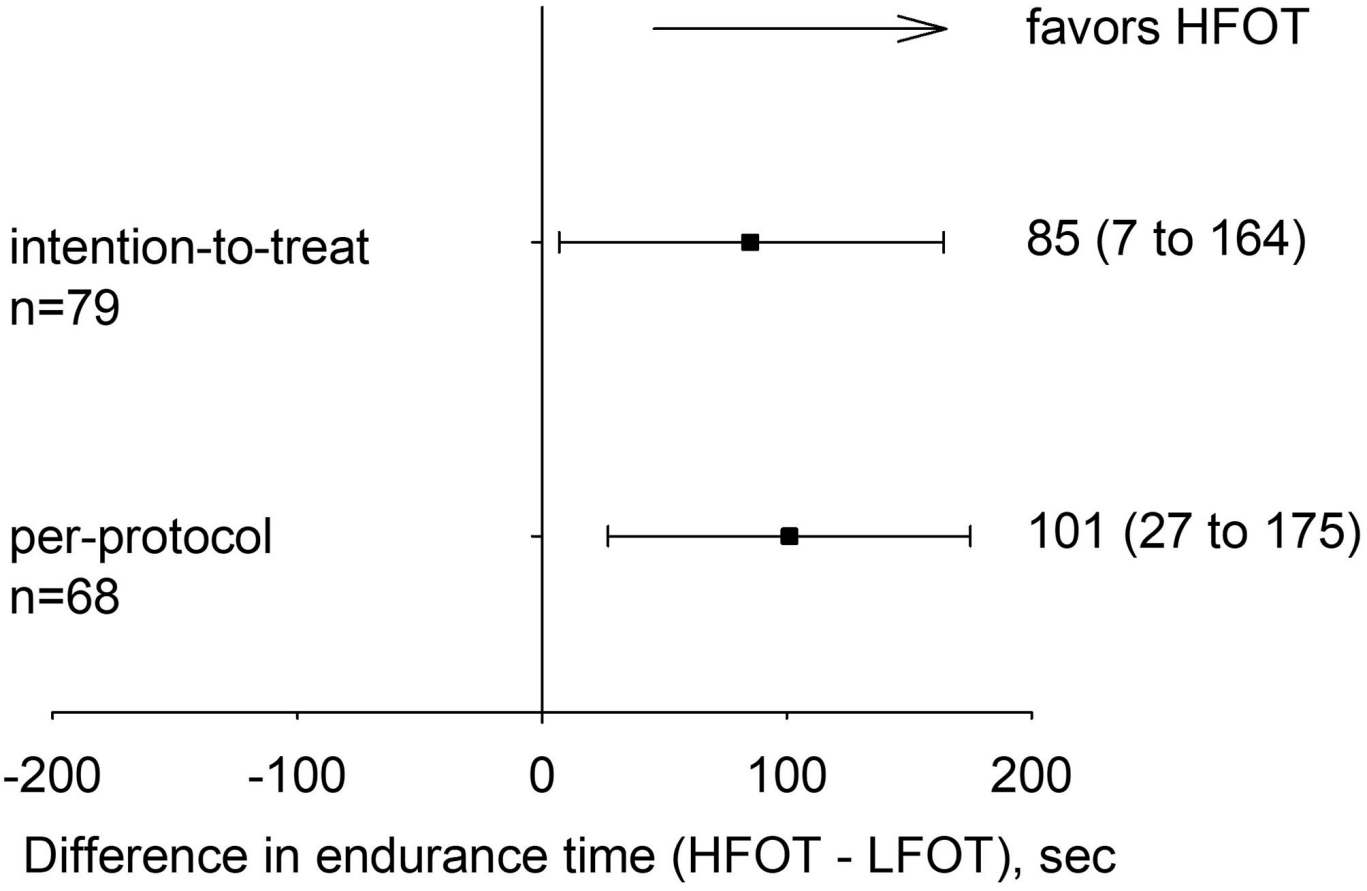
Primary outcome: difference with 95% confidence intervals in cycling endurance time between tests on high-flow oxygen therapy (HFOT) and low-flow oxygen therapy (LFOT) for the intention-to-treat and per-protocol analyses.

In the intention-to-treat analysis, mixed linear regression analysis with intervention (HFOT, LFOT) and allocation sequence as predictors revealed a mean ± SD endurance time with HFOT of 773 ± 471 s and with LFOT of 688 ± 463 s. Thus, HFOT improved endurance time by a mean of 85 s (95% CI 7 to 164, *P* = 0.034) or 13% (1 to 28) corresponding to an effect size of 0.20 (95% CI: 0.02 to 0.38) (Figure 2, Table 2). Adjusted analysis did not reveal any significant effect of age (coefficient −5, 95% CI −16 to 6), sex (coefficient 80, 95% CI −115 to 276), or FEV_1_% predicted (coefficient 3, 95% CI −7 to 14) on the difference between tests with HFOT and LFOT. In the per-protocol analysis, endurance time was increased by HFOT by a mean of 101 s (95% CI: 27 to 175, *P* = 0.007) (Table 2) corresponding to an effect size of 0.26 (95% CI: 0.07 to 0.44).

**Table 2:** Main outcomes.

	**Low-flow oxygen therapy, LFOT**			**High-flow oxygen therapy, HFOT**			
	**Rest**	**End- exercise**	**Difference end-exercise minus rest**	**Rest**	**End-exercise**	**Difference end-exercise minus rest**	**Difference HFOT minus LFOT at end- exercise**
Endurance time, intention-to-treat analysis, s	NA	688 ± 463		NA	773 ± 471		85 (7 to 164)^*^
Endurance time, per-protocol analysis, s	NA	612 ± 452		NA	713 ± 452		101 (27 to 175)^*^
Breath rate, 1/min	16.9 ± 6.4	33.4 ± 6.4	16.5 (14.9 to 18.1)^*^	15.8 ± 6.4	32 ± 6.4	16.3 (14.7 to 17.8)^*^	−1.4 (−2.9 to 0.2)
Heart rate, 1/min	79 ± 16	137 ± 16	59 (55 to 63)^*^	78 ± 16	135 ± 16	57 (54 to 61)^*^	−2 (−6 to 2)
Heart rate reserve, 1/min	84 ± 16	25 ± 16	−59 (−63 to −55)^*^	85 ± 16	27 ± 16	−57 (−61 to −54)^*^	2 (−2 to 6)
SpO_2_, %	97 ± 0	96 ± 0	−1 (−1 to 0)^*^	98 ± 0	98 ± 0	−1 (−1 to 0)^*^	2 (2 to 2)^*^
Arterial pH^†^	NA	7.33 ± 0.00	−0.07 (−0.08 to −0.06)^*^	NA	7.34 ± 0.1	−0.06 (−0.07 to −0.05)^*^	0.01 (−0.01 to 0.02)
PaCO_2_, kPa^†^	NA	4.9 ± 0.8	−0.5 (−0.6 to −0.4)^*^	NA	4.8 ± 0.8	−0.7 (−0.8 to −0.5)^*^	−0.1 (−0.3 to 0.03)
PaO_2_, kPa^†^	NA	14.4 ± 3.2	5.0 (4.0 to 5.9)^*^	NA	21.6 ± 3.2	12.1 (11.1 to 13.1)^*^	7.1 (5.8 to 8.5)^*^
Arterial ${\text{HCO}}_{3}^{-}$, mmol/l ^†^	NA	19.3 ± 2.4	−5.6 (−6.2 to −5.0)^*^	NA	19.2 ± 2.4	−5.7 (−6.3 to −5.1)^*^	−0.1 (−0.9 to 0.7)
SaO_2_, %^†^	NA	97 ± 2	4 (3 to 4)^*^	NA	99 ± 2	5 (5 to 5)^*^	1 (1 to 2)^*^
Lactate, mmol/l^†^	NA	6.7 ± 2.1	5.7 (5.0 to 6.3)^*^	NA	6.4 ± 2.2	5.4 (4. 8 to 6.1)^*^	−0.2 (−1.1 to 0.7)
Borg dyspnea	0.3 ± 1.6	4.0 ± 1.6	3.7 (3.2 to 4.1)^*^	0.2 ± 1.6	3.3 ± 1.6	3 (2.6 to 3.5)^*^	−0.8 (−1.2 to −0.3)^*^
Borg leg fatigue	0.4 ± 1.6	5.3 ± 1.6	4.9 (4.5 to 5.4)^*^	0.4 ± 1.6	5.4 ± 1.6	5 (4.5 to 5.4)^*^	0 (−0.4 to 0.5)
Treatment comfort, mm VAS	NA	58 ± 17		NA	58 ± 20		0 (−6 to 6)

*Means ± SD, mean differences (95% confidence intervals). For the primary outcome, endurance time, results are presented for intention-to-treat (n = 79) and per-protocol analyses (n = 68). For all other outcomes, results obtained with the corresponding treatment are presented for the per protocol analysis*.

*SpO_2_, pulse oximetry. NA, not applicable*.

† *Resting arterial blood gas analyses were obtained once per patient while breathing ambient air, results are listed in Table 1. All other outcomes were obtained while patients were breathing the corresponding treatment. Treatment comfort was rated on a visual analog scale (VAS, 100 mm in length)*.

* *Difference P < 0.05*.

Secondary outcomes at rest and end-exercise are presented in Table 2. In tests with HFOT and LFOT, there were similar exercise-induced increases in breath rate and in heart rate. A metabolic acidosis associated with a reduction in PaCO_2_ was observed. Compared to resting arterial blood gas analysis during ambient air breathing, there was a significantly greater increase in PaO_2_ and SaO_2_ at end-exercise with HFOT than with LFOT with mean differences of 7.1 kPa (95% CI 5.8 to 8.5, *P* < 0.001 and 1% (1 to 2, *P* < 0.001).

**Figure 3:**
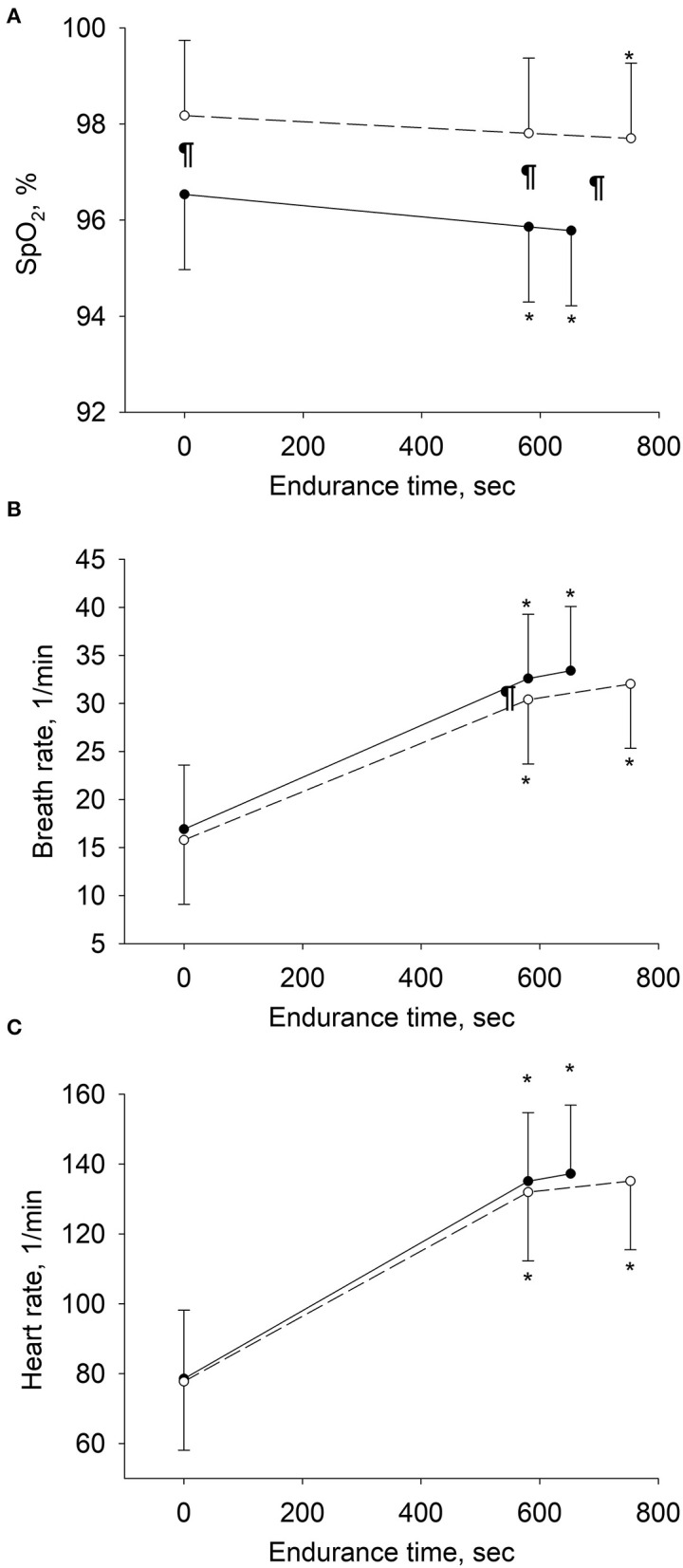
Changes in physiologic variables over the course of exercise in per-protocol analyses. Means and SD bars are shown for values at rest, at isotime (i.e., end-exercise time in tests with shorter endurance and corresponding time in tests with longer endurance), and at end-exercise. Open circles represent tests with high-flow, closed circles with low-flow oxygen therapy. **(A–C)** Depict pulse oximetry (SpO_2_), breath rate and heart rate. *P < 0.05 vs. rest within same treatment, ^¶^*P* < 0.05 high-flow vs. low-flow oxygen therapy at corresponding stage of exercise.

Changes in physiologic variables over the course of exercise tests from rest to isotime and end-exercise are displayed in Figure 3. HFOT reduced the respiratory rate at isotime by 2.2 breaths/minute (95% CI: 0.7 to 3.7, *P* = 0.006) and improved SpO_2_ by 2% (2 to 2, *P* < 0.001).

Patients perceived less dyspnea at end-exercise under HFOT (−0.8 points Borg CR10 scale, 95% CI: −1.2 to −0.3, *P* = 0.044). Comfort with treatments was rated similarly with both modalities (mean difference 0.1%, 95% CI: −6.4 to 6.2).

## Discussion

The current randomized cross-over trial is the first to evaluate the effect of HFOT on exercise performance in comparison to LFOT in mildly hypoxemic patients with moderate to severe COPD. The main findings are an improvement in endurance time by 13% associated with a higher arterial oxygen saturation, a lower respiratory rate at corresponding isotime and less dyspnea at end-exercise.

The use of LFOT by nasal cannula has been shown to improve both endurance time and peak work rate in patients with non-hypoxemic COPD when used repeatedly during exercise training programs over many weeks (9, 32). However, the discomfort associated with LFOT by mucosal drying and a variable or reduced FiO_2_ due to mouth breathing during exercise hamper its acceptance and efficacy. HFOT with humidified, heated air was expected to address these shortcomings but its effect on endurance time has not been conclusively studied yet. Thus, in a randomized trial investigating effects of nasal high-flow breathing of ambient air or oxygen-enriched air, Cirio et al. (19) demonstrated that HFOT (60 l/min) improved exercise performance in 12 patients with stable severe COPD (mean FEV_1_ 35% predicted, mean PaO_2_ 9.7 kPa) and ventilatory limitation compared to breathing with a Ventury mask with individually variable FiO_2_ set to maintain oxygen saturation >88%. Since oxygen was administered in 8 of 12 patients due to exercise-induced hypoxemia both during the nasal high-flow and low-flow control tests, the specific effects of HFOT could not be assessed. Another randomized trial in 19 patients with severe to very severe COPD (mean FEV_1_ 29% predicted, mean PaO_2_ 9.2 kPa) recovering from an exacerbation, revealed no significant improvement in endurance time with nasal high-flow, but comparison to low-flow was hampered since variable amounts of supplemental oxygen were administered to maintain SpO_2_ ≥90% both during high-flow and control tests (20). Compared to the cited studies, the degree of airflow obstruction in participants of the current trial was less severe (Table 1), but the mean resting PaO_2_ was similarly reduced compared to the predicted mean PaO_2_ for normals (33, 34).

The current study is the first investigating the effects of HFOT compared to LFOT in a methodologically sound randomized trial. The increase in cycling endurance time by a mean of 85 s achieved by a single application of HFOT vs. LFOT is considerable as it exceeds the minimal clinically important difference in endurance time suggested for COPD patients (28). Since constant-load exercise training improves aerobic exercise capacity and muscular function in COPD patients (6), longer exercise training under HFOT may favorably affect the individually targeted outcomes during a training program and thereby improve exercise tolerance and quality of life. In the current study, the improvement in performance was related to a higher arterial oxygen saturation under HFOT both at isotime and at end-exercise compared to LFOT. Correspondingly, in healthy individuals and in patients with COPD (35) or precapillary pulmonary hypertension (36), breathing hyperoxic air improved pulmonary gas exchange, reduced ventilatory work and resulted in enhanced exercise endurance (37). Even though the PaO_2_ and SaO_2_ at end-exercise were higher with HFOT vs. LFOT in COPD patients in the current study, this did not promote a rise in PaCO_2_ under HFOT (**Table 2**).

The higher PaO_2_ at end-exercise during HFOT was associated with less dyspnea sensation compared to LFOT which may have contributed to the prolongation of endurance with HFOT. In turn, the leg fatigue sensation, the levels of lactate and heart rate reserve at end-exercise were comparable between the two therapies suggesting that respiratory limitations rather than circulatory or peripheral muscle limitations were the main determinants of endurance although this could not be directly assessed in the current protocol. *Post-hoc* exploratory regression analysis performed to identify potential predictors of a favorable response to HFOT did not reveal statistically significant effects of age, sex, severity of airflow obstruction or body mass index.

Although a tendency for a greater reduction of PaCO_2_ under HFOT than under LFOT was observed, this difference was not statistically significant (Table 2) and we therefore have no evidence of a reduction in dead space ventilation by HFOT as observed in certain previous studies (11). Perhaps our study was underpowered to detect small differences in PaCO_2_. Moreover, patients could not take advantage of the PEEP-effect of HFOT, which requires breathing with the mouth closed (38, 39), which was not feasible during the exercise.

Contrary to the study of Prieur et al. (20) in which about half of the COPD patients recovering from an exacerbation tolerated HFOT poorly (flow rate of 60 L/min and temperature of 31°C), patients in the current study perceived the treatment fairly comfortable.

The current trial included only mildly hypoxemic, non-hypercapnic patients with moderate to severe COPD. Therefore, extrapolations to patients with milder or very severe COPD should be done with caution even though FEV_1_ was not a predictor of the treatment effect in regression analysis. Blinding of patients and investigators for the interventions was not feasible as the flow rate of 60 L/min and the flow rate of 3 L/min could be easily noticed. However, patients had no experience of any of the two treatments and the study hypothesis was not disclosed. We chose a HFOT setting of 60 L/min, temperature of 31°C and FiO_2_ of 0.45 to investigate the combined effects of high flow rate and mild hyperoxia. Choosing different settings of HFOT may have resulted in other effects. We administered LFOT via conventional nasal cannula at a rate of 3 l/min as the control intervention because this is the standard way of oxygen supplementation in clinical practice (9, 18, 21). Although higher oxygen flow rates of, for example, 5–15 l/min by conventional nasal cannula, might have increased the FiO_2_, an exact matching to the FiO_2_ administered during HFOT would not have been feasible and might have caused nasal mucosal irritation and discomfort. Whether the improvement in endurance time by HFOT was due to a higher FiO_2_ or a higher rate of nasal flow can therefore not be differentiated by our data.

In conclusion, our study showed an improvement in cycling endurance time by HFOT in patients with stable, mildly hypoxemic, moderate to severe COPD. HFOT was associated with a lower breath rate at isotime, a higher arterial oxygen saturation and less dyspnea and it was well-tolerated. These results are applicable for many COPD patients seen in daily practice and indicate that patients may benefit from HFOT during exercise training.

## Data Availability Statement

The raw data supporting the conclusions of this article will be made available by the authors, without undue reservation.

## Ethics Statement

The studies involving human participants were reviewed and approved by Ethics Committee of the National Center for Cardiology and Internal Medicine, Bishkek, Kyrgyz Republic. The patients/participants provided their written informed consent to participate in this study.

## Author Contributions

KB and MF contributed to the conception and design of the study, data collection, analysis, interpretation, and drafting the article. LM, SS, SB, MM, US, NM, AA, SA, and AM contributed to data collection, analysis, and interpretation of data. TS, SU, and KEB contributed to obtaining funding, to the conception and design of the study, acquisition, analysis, and interpretation of data. All authors critically revised the manuscript for important intellectual content, they approved the version to be published and all agree to be accountable for all aspects of the work in ensuring that questions related to the accuracy or integrity of any part of the work are appropriately investigated and resolved.

## Conflict of Interest

KEB reports grants to his institution from the Swiss National Science Foundation, and the Bockhoff Foundation. The high-flow equipment was provided by Fisher & Paykel Healthcare, Switzerland; the oxygen concentrators were provided by Philips AG Respironics, Switzerland. The remaining authors declare that the research was conducted in the absence of any commercial or financial relationships that could be construed as a potential conflict of interest.
